# Severe Leptospirosis (Weil’s Disease) Presenting With Fulminant Alveolar Hemorrhage and Multiorgan Failure: A Case Report

**DOI:** 10.7759/cureus.113916

**Published:** 2026-08-03

**Authors:** Alexis Loulas, Tuan Tran, Virginie Meurant

**Affiliations:** 1 Emergency, Université Libre de Bruxelles, Brussels, BEL; 2 Emergency Medicine, Centre Hospitalier Universitaire Tivoli, La Louvière, BEL

**Keywords:** acute respiratory distress syndrome (ards), alveolar hemorrhage, hemorrhagic fever, leptospirosis, zoonosis

## Abstract

We report the case of a 50-year-old man admitted to the emergency department with fever, asthenia, and diarrhea, who developed multiorgan failure and acute respiratory distress syndrome (ARDS) due to a massive, abrupt-onset alveolar hemorrhage secondary to leptospirosis infection. Leptospirosis is a zoonosis mainly transmitted by rats, with an increasing incidence in Belgium and the rest of Western Europe. The wide spectrum of symptoms and variability in disease severity make this diagnosis particularly challenging. In this case, initial serological testing, including the microscopic agglutination test (MAT), was negative, leading to a diagnostic delay despite a severe clinical presentation, and the diagnosis was only confirmed after repeated MAT testing. This clinical case describes a severe presentation and highlights the importance of thorough history-taking, including information provided by the patient's relatives, together with repeated etiological testing when clinical suspicion persists. Following intensive care management, the patient showed progressive clinical improvement and was ultimately discharged from the intensive care unit and from the hospital.

## Introduction

Leptospirosis is an emerging zoonotic infection caused by pathogenic spirochetes of the genus *Leptospira*, primarily transmitted through exposure to water or soil contaminated with the urine of infected rodents [1]. Although classically associated with tropical and subtropical regions, its incidence has been increasing in temperate areas, including Belgium and other parts of Western Europe. Global estimates suggest that leptospirosis causes approximately one million severe cases and nearly 60,000 deaths annually worldwide [2]. The World Health Organization (WHO) estimates an annual incidence of approximately 0.1 to one case per 100,000 population in temperate climates, rising to 10-100 cases per 100,000 in humid tropical regions, and exceeding 100 per 100,000 among high-exposure groups or during outbreaks [3]. In Belgium, national surveillance data reported an average of approximately 15 confirmed cases per year between 2000 and 2010, rising to around 30 cases per year between 2010 and 2020, with 41 confirmed cases recorded in 2024, according to the 2024 epidemiological surveillance report on leptospirosis (*Leptospira* spp.) by Lernout et al. [4]. This trend has been linked in part to changing patterns of recreational freshwater exposure, urban rodent infestation, and improved diagnostic awareness. The clinical presentation is highly variable, ranging from a mild, flu-like illness to severe, life-threatening multiorgan involvement.

The most severe form, known as Weil's disease, is characterized by jaundice, acute kidney injury, hemorrhagic manifestations, and, in some cases, acute respiratory distress syndrome (ARDS) related to pulmonary hemorrhage. Among these manifestations, severe pulmonary hemorrhagic syndrome (SPHS) with diffuse alveolar hemorrhage represents a relatively rare but particularly lethal complication of leptospirosis, with reported mortality rates frequently exceeding 50% in published series, despite intensive care management. Pulmonary involvement may affect up to 70% of severe cases worldwide [5] and is distinctive in its capacity to progress from subtle radiographic changes to massive, life-threatening hemoptysis and refractory hypoxemia within hours, a trajectory that distinguishes it from most other causes of ARDS, and that is directly illustrated by the abrupt clinical deterioration described in this report. In some cohorts, pulmonary involvement has emerged as a major contributor to death in severe leptospirosis, sometimes surpassing classical hepatic or renal failure as the predominant cause of mortality [6].

Diagnosis can be challenging due to the nonspecific nature of symptoms and their overlap with other infectious and autoimmune conditions. Early recognition and prompt management are essential to improving patient outcomes. Here, we report the case of an unemployed 50-year-old man who presented with fulminant alveolar hemorrhage and multiorgan dysfunction as the initial manifestation of leptospirosis. This case is noteworthy for the occurrence of severe pulmonary hemorrhagic syndrome in a non-tropical, temperate setting, the initial failure of serological testing to establish the diagnosis, and the crucial role of collateral exposure history (freshwater immersion in an urban canal) in raising diagnostic suspicion. In addition, the case illustrates how repeated microscopic agglutination testing (MAT) is essential when initial results are negative but clinical suspicion remains high, and it underscores the need for intensivists and internists in Western Europe to consider leptospirosis in patients presenting with unexplained diffuse alveolar hemorrhage and multiorgan failure. Notably, severe leptospirosis can be clinically indistinguishable from other undifferentiated febrile zoonoses, particularly hantavirus infection, which shares a similar rodent-associated transmission route and can produce an overlapping presentation of fever, thrombocytopenia, and multiorgan involvement; this diagnostic overlap, discussed further below, constituted a central challenge in the present case.

Compared with previously published reports of leptospirosis-associated diffuse alveolar hemorrhage, which most often describe occupational, agricultural, or recreational exposure in endemic or rural settings, the present case is distinguished by an incidental, brief freshwater immersion in an urban canal in Western Europe, an initially anicteric presentation, and a full recovery achieved with lung-protective ventilation and multiorgan support alone, without recourse to extracorporeal membrane oxygenation, corticosteroids, or plasma exchange. The exposure history - the fall into the canal 48 hours before symptom onset - is deliberately presented later in this report, at the point in the clinical course when it was actually obtained from the patient's family, rather than earlier in the initial history. This narrative structure is intentional: it allows the reader to first engage with the presentation, examination findings, and initial investigations exactly as the treating team encountered them, without the benefit of the exposure history, before the collateral information reframes the diagnostic reasoning. This structure is designed to reproduce, for teaching purposes, the real-time diagnostic uncertainty faced at the bedside, and it reinforces the report's central educational point on the value of collateral history in critically ill patients.

## Case presentation

A 50-year-old Caucasian man presented to the emergency department of Centre Hospitalier Universitaire (CHU) Tivoli in La Louvière, Belgium, with asthenia and diarrhea. Over the preceding four days, he reported subjective fever without documented temperature, associated with chills, marked fatigue, and approximately four daily episodes of non-bloody, watery diarrhea. He expressed concern about progressively worsening fatigue occurring even outside febrile episodes and accompanied by presyncopal symptoms. He reported a subjective sensation of “intoxication.” On examination, this correlated with objective bradypsychia, dysarthria, somnolence, and impaired concentration. He denied headache, dyspnea, cough, chest pain, palpitations, abdominal pain, or urinary symptoms.

The patient had a cardiovascular history notable for arterial hypertension, hypercholesterolemia, ischemic heart disease (left anterior descending artery angioplasty in 2005), ischemic stroke of the posterior fossa (2015), and atrial fibrillation. Additional relevant history included sleep apnea syndrome and bipolar disorder. He reported no known allergies or recent contact with individuals with infectious diseases. He admitted to alcohol and tobacco use but was unable to quantify his consumption and stated that he had not consumed alcohol on the day of admission. He lived alone in an urban environment with his dog, which was reported to be in good health; the dog's vaccination status was unknown. He had not traveled within the previous six months.

Clinical examination

On initial examination, his temperature was 39 °C, heart rate 80 beats per minute, blood pressure 70/50 mmHg, respiratory rate 18 breaths per minute, and oxygen saturation 99% on room air. His body mass index (BMI) was 21.0 kg/m² (60 kg for 1.69 m). The jugular veins were flat, mucous membranes were dry, and central capillary refill time was less than three seconds. The patient was oriented to time and place; however, his speech was disorganized and mildly incoherent. The abdomen was distended but soft and non-tender. Cardiopulmonary auscultation was unremarkable, with no focal neurological deficit and no lymphadenopathy. Skin examination revealed pallor with mild jaundice, without mucocutaneous lesions or rash.

There was no conjunctival suffusion, no skin rash, and no clinical hepatomegaly or splenomegaly. However, the patient reported diffuse myalgia, and mild muscle tenderness was elicited on palpation of the calves and proximal limb muscles.

He received placement of an indwelling urinary catheter, and urine output remained nil throughout his stay in the emergency department.

During initial management, the patient abruptly developed dyspnea that progressed within minutes to a respiratory rate of 60 breaths per minute, accompanied by signs of severe respiratory distress with global chest retractions. Crackles, initially localized at the right lung base, rapidly became diffuse. Oxygen saturation decreased to 70% despite a non-rebreathing mask. Concomitantly, he developed generalized mottling and a prolonged capillary refill time exceeding four seconds.

Additional investigations

Arterial blood gas analysis performed on admission without oxygen supplementation showed a PaO₂/FiO₂ ratio greater than 347. The initial microbiological work-up included a nasopharyngeal swab for COVID-19, urine analysis with *Legionella* antigen testing, and two sets of blood cultures; stool cultures were not obtained. Laboratory findings demonstrated a marked inflammatory response with C-reactive protein of 100.9 mg/L (normal: <0.5 mg/L), lymphopenia (280/mm³; normal: 1,000-4,000/mm³), thrombocytopenia (97,000/mm³; normal: 150,000-400,000/mm³), acute kidney injury (urea 169 mg/dL; normal: 15-45 mg/dL, creatinine 2.96 mg/dL; normal: 0.67-1.17 mg/dL), creatine kinase of 223 U/L (normal: 30-200 U/L), elevated lactate dehydrogenase (1034 U/L; normal: 125-220 U/L), aspartate aminotransferase elevation (72 U/L; normal: 0-40 U/L), elevated gamma-glutamyl transferase (106 U/L; normal: 8-61 U/L), direct hyperbilirubinemia (2.31 mg/dL; normal: 0.0-0.3 mg/dL), international normalized ratio (INR) of 1.33 (normal: 0.8-1.2) in the context of apixaban treatment, hyperkalemia (5.2 mmol/L; normal: 3.5-5.0 mmol/L), and hyperphosphatemia (1.69 mmol/L; normal: 0.8-1.5 mmol/L). Procalcitonin, D-dimers, fibrinogen, and ferritin were not initially measured. Initial microbiological tests were negative.

**Figure 1:**
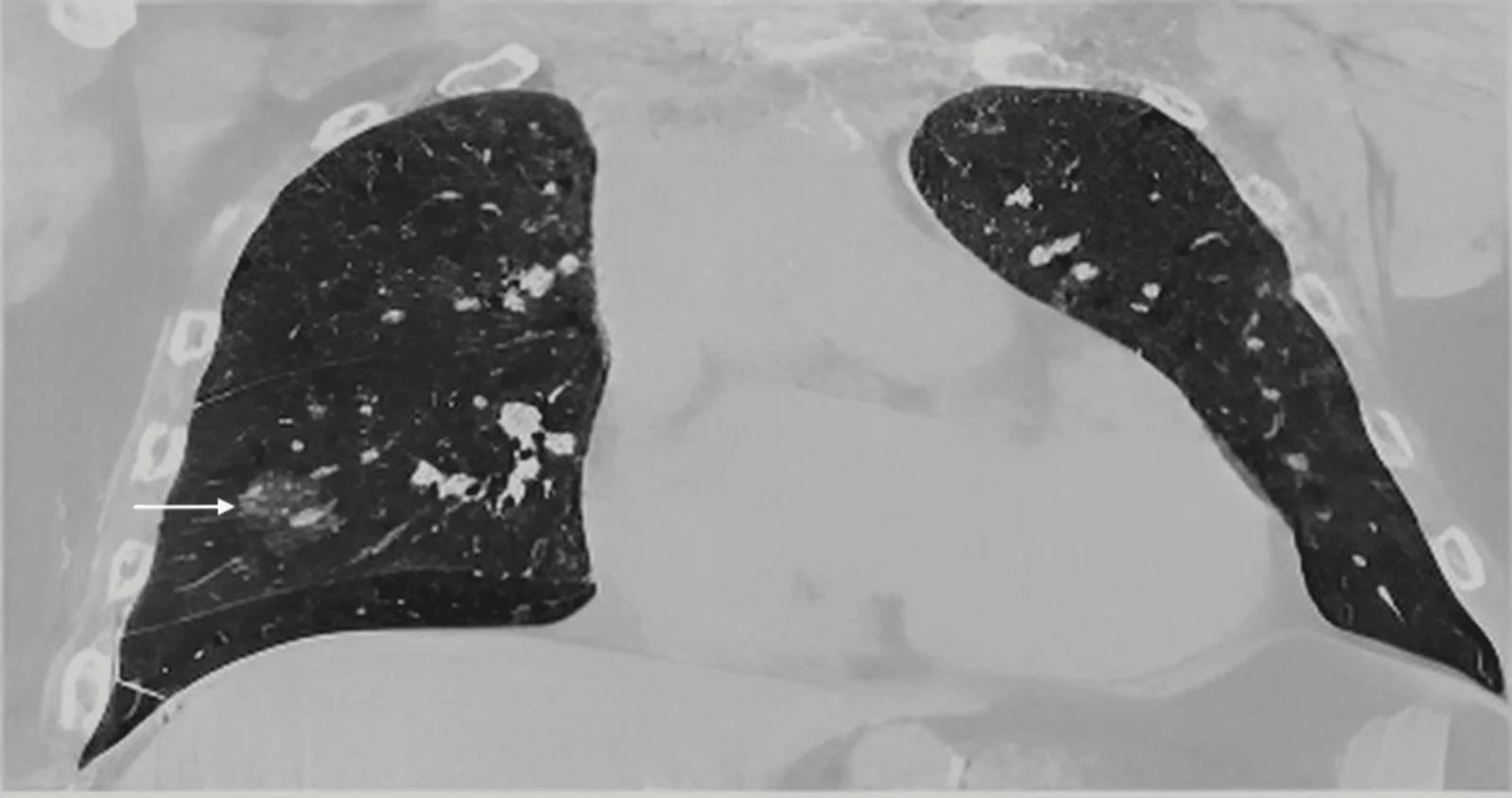
Non-contrast chest CT, coronal view. The arrow indicates a nodular lesion in the right lower lobe.

Non-contrast computed tomography of the brain, chest, and abdomen revealed no acute intracranial abnormalities and no significant pulmonary or mediastinal findings. However, a 26 mm left adrenal nodule was incidentally identified. Figure 1 shows the initial imaging. A nodular lesion in the right lower lobe was later identified; this finding had not been mentioned in the initial radiology report, which was issued in the emergency setting, where a small pulmonary nodule may be overlooked when the radiologist is focused on identifying a cause for profound shock in a patient whose initial presentation was dominated by diarrhea and confusion.

Therapeutic management

This 60 kg patient received a second peripheral intravenous line and 2 liters of Plasma-Lyte over two hours. Empiric antibiotic therapy was initiated with 2 g amoxicillin-clavulanic acid and 1.5 g amikacin administered as intravenous boluses. Amoxicillin-clavulanate was selected empirically as broad-spectrum coverage for a presumed infectious source, while amikacin was added given the context of shock. At the time of initial treatment, no element of the clinical history or presentation pointed toward leptospirosis, a diagnosis that would otherwise have favored ceftriaxone as first-line empiric therapy.

An additional liter of Plasma-Lyte over 30 minutes was infused during placement of a right radial arterial line and a right internal jugular central venous line.

**Figure 2:**
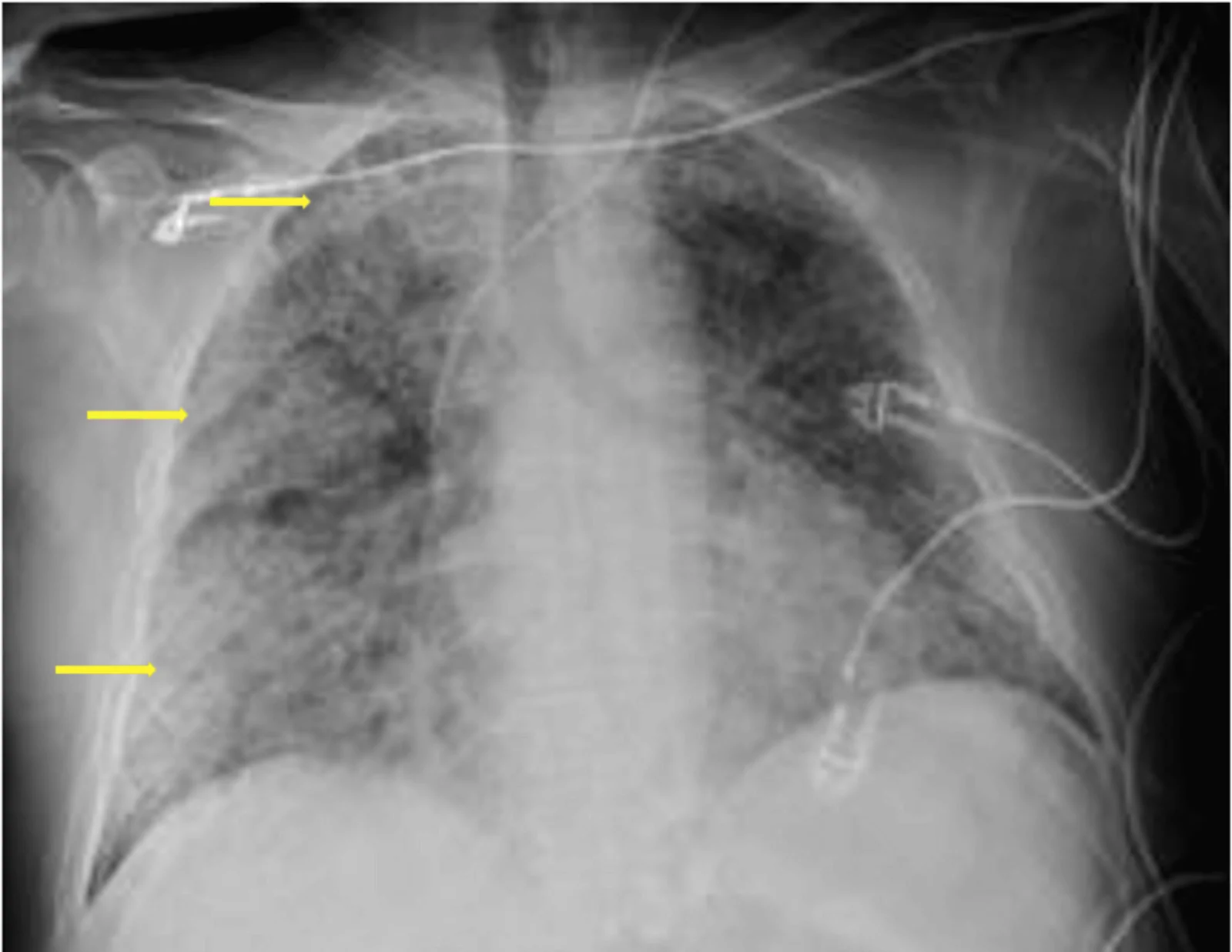
Chest X-ray, anteroposterior (AP) view, showing acute respiratory distress syndrome (ARDS). Diffuse alveolar and interstitial infiltrates distributed peripherally in both lungs, more pronounced on the right side, as indicated by the yellow arrows.

Despite these measures, he developed rapidly progressive acute respiratory failure requiring endotracheal intubation. Pre-intubation arterial blood gas analysis under a non-rebreathing mask (FiO₂ 60%) showed a PaO₂/FiO₂ ratio of 158, consistent with moderate ARDS (Figure 2). Orotracheal suctioning yielded a large amount of fresh, frothy blood, consistent with alveolar hemorrhage.

The patient was transferred to the intensive care unit (ICU), where management included high-dose norepinephrine infusion up to 2 mg per hour, mechanical ventilation with high positive end-expiratory pressure, neuromuscular blockade, continuous renal replacement therapy, and escalation of antimicrobial therapy to a third-generation cephalosporin. A lung-protective ventilation strategy was applied, with PEEP progressively increased up to 18 cmH₂O and tidal volume reduced to 260 mL (approximately 4 mL/kg of ideal body weight). Prone positioning was not required. The clinical severity peaked on the fourth day of ICU admission, and the patient was successfully weaned from mechanical ventilation twelve days later. Corticosteroids, tranexamic acid, blood transfusion, plasma exchange, and extracorporeal membrane oxygenation (ECMO) were not administered, as the patient's respiratory and hemodynamic status progressively improved with supportive intensive care measures alone.

**Figure 3:**
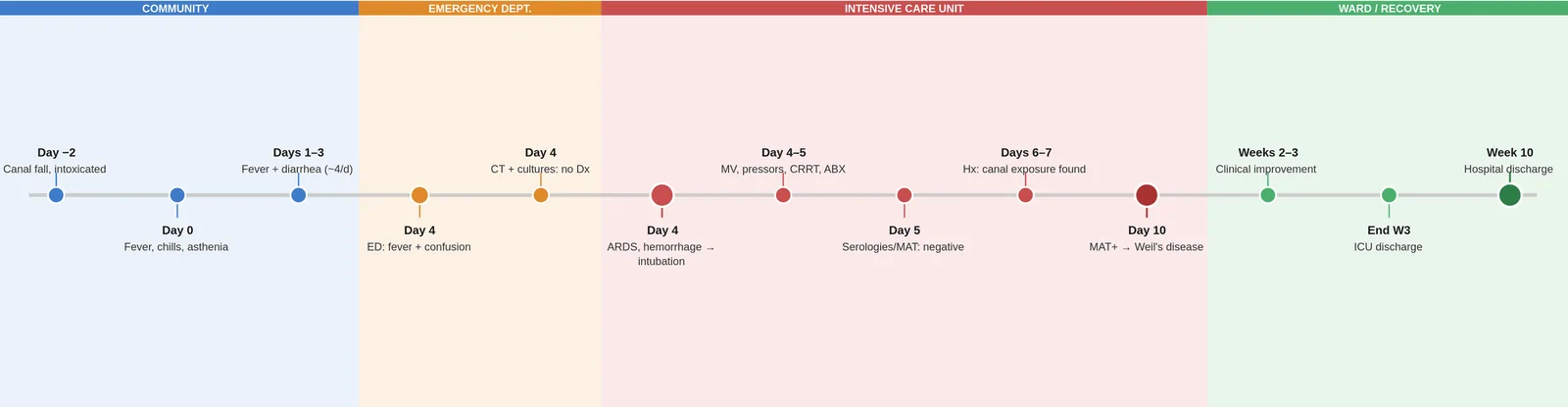
Timeline of the clinical course ABX: antibiotics; ARDS: acute respiratory distress syndrome; CRRT: continuous renal replacement therapy; CT: computed tomography; Dx: diagnosis; ED: emergency department; Hx: history; ICU: intensive care unit; MAT: microscopic agglutination test; MV: mechanical ventilation; W3: week 3

Subsequently, collateral history obtained from a family member revealed that the patient had fallen into the Canal du Centre in La Louvière while acutely intoxicated approximately 48 hours before symptom onset. The patient's clinical course gradually improved, allowing ICU discharge after three weeks and hospital discharge 10 weeks after admission (Figure 3 summarizes the clinical timeline). At discharge, the patient returned home without additional assistance, suggesting preserved functional status and quality of life. However, he did not attend the scheduled six-month pulmonology and nephrology follow-up consultations, so formal long-term data on pulmonary function, renal recovery, and neurological status could not be obtained.

Differential diagnosis

This patient presented with fever, asthenia, and diarrhea, along with lymphopenia, thrombocytopenia, stage 3 Kidney Disease: Improving Global Outcomes (KDIGO) acute kidney injury, and direct hyperbilirubinemia. Clinical findings included encephalopathy and ARDS with alveolar hemorrhage. Table 1 summarizes the main differential diagnoses considered, together with the clinical and paraclinical findings supporting or opposing each.

**Table 1:** Differential diagnosis of fever with multiorgan failure and diffuse alveolar hemorrhage DIC: disseminated intravascular coagulation; CT: computed tomography; BAL: bronchoalveolar lavage; PCR: polymerase chain reaction; ANCA: antineutrophil cytoplasmic antibodies; GBM: glomerular basement membrane; GPA: granulomatosis with polyangiitis; MPA: microscopic polyangiitis; EGPA: eosinophilic granulomatosis with polyangiitis; ENT: ear, nose, and throat; MAT: microscopic agglutination test

Diagnosis	Supporting findings	Opposing findings
Severe bacterial sepsis with DIC	Profound shock, thrombocytopenia, coagulopathy	Sterile blood cultures (×2); no identifiable source
Legionella infection	Potential for severe pneumonia with multiorgan involvement	Negative urinary antigen; no pulmonary/mediastinal abnormality on CT
Invasive pneumococcal disease	Leading cause of severe community-acquired sepsis	No respiratory or meningeal focus; negative blood cultures
Influenza / COVID-19	Can cause multiorgan failure; influenza rarely causes alveolar hemorrhage	Negative nasopharyngeal and BAL PCR for influenza A/B and SARS-CoV-2
Rickettsiosis	Can present with severe multisystem involvement	No skin lesions or eschar
Q fever (Coxiella burnetii)	Flu-like illness, digestive symptoms, hepatitis	Respiratory involvement typically atypical pneumonia, not alveolar hemorrhage
Pulmonary–renal syndrome (anti-GBM, GPA, MPA, EGPA, cryoglobulinemia)	Alveolar hemorrhage with renal involvement	Negative ANCA, anti-GBM antibodies, cold agglutinins; no asthma, eosinophilia, or ENT involvement; less abrupt onset expected
Macrophage activation syndrome (secondary to sepsis)	Multiorgan failure in septic context	Negative hemophagocytosis on bone marrow aspiration
Hantavirus infection	Overlapping presentation with leptospirosis; rodent exposure	Repeated serologies and RT-PCR negative
Leptospirosis (final diagnosis)	Freshwater immersion 48h before onset; multiorgan failure, ARDS with alveolar hemorrhage	Initial MAT negative — diagnosis confirmed only on repeat testing at day 10

Ultimately, the differential diagnosis focused on hantavirus infection versus leptospirosis.

Results and follow-up

The absence of antineutrophil cytoplasmic antibodies (ANCA), anti-GBM antibodies, cold agglutinins, asthma, eosinophilia, ENT involvement, and hemophagocytosis on bone marrow aspiration made non-infectious etiologies unlikely. Nasopharyngeal swabs and bronchoalveolar lavage (BAL) polymerase chain reaction (PCR) testing were negative for influenza A, influenza B, and SARS-CoV-2. Serological testing for Rickettsia and Coxiella was negative, as were serologies for hepatitis A, B, C, and E, human immunodeficiency virus (HIV), and cytomegalovirus/Epstein-Barr virus (CMV/EBV) PCR.

**Figure 4:**
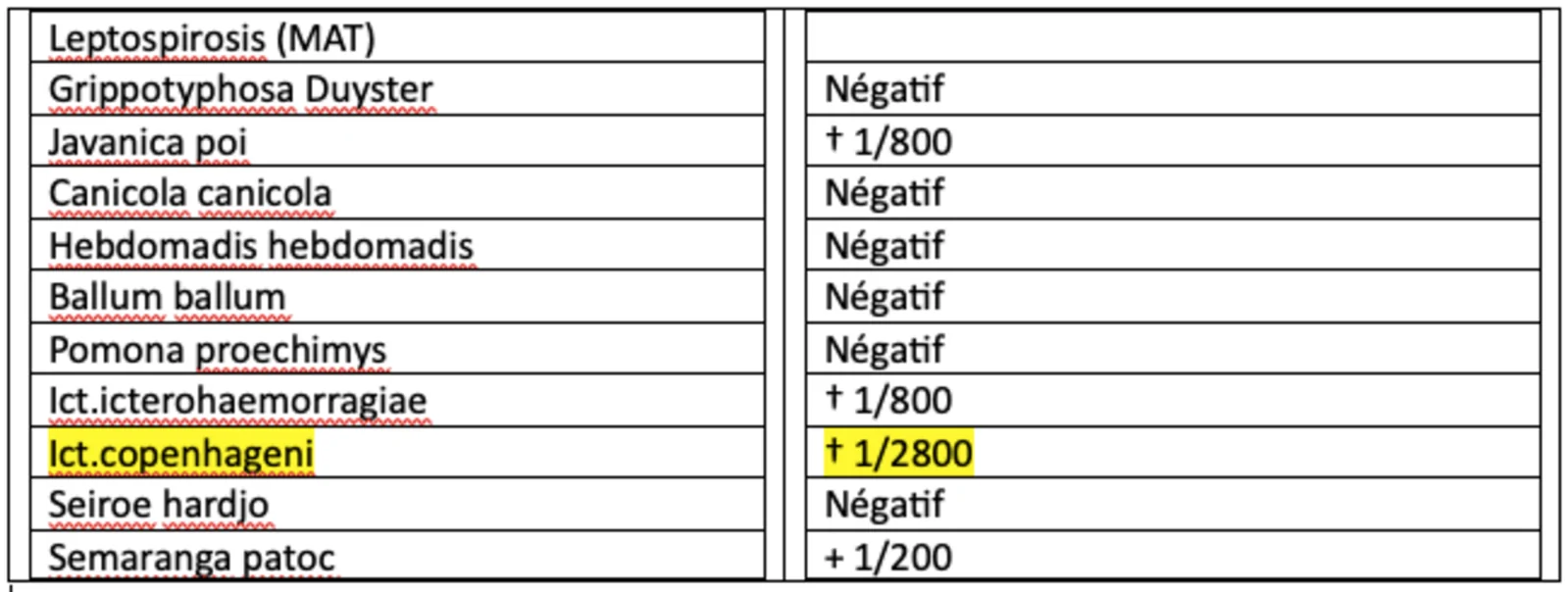
Results of the microscopic agglutination test (MAT) The significant result is highlighted: serovar Icterohaemorrhagiae Copenhageni. In this French-language laboratory report, "Négatif" indicates a negative result, a plus sign (+) indicates positive but non-significant reactivity, and a dagger symbol (†) indicates a positive and significant result.

Repeated hantavirus serologies and reverse transcription PCR (RT-PCR) were also negative. Microscopic agglutination test (MAT) for *Leptospira*, performed the day after admission, was initially negative, and blood and urine cultures showed no growth. Repeat MAT testing was performed on day 10, once collateral history revealed freshwater immersion in an urban canal approximately 48 hours before symptom onset, raising strong clinical suspicion for leptospirosis despite the initial negative result. This decision was further supported by the well-established kinetics of the humoral response in leptospirosis: MAT antibodies typically become detectable only toward the end of the first week of illness and may remain negative if testing is performed earlier, during the leptospiremic phase that precedes seroconversion. An initial negative MAT therefore does not rule out the diagnosis when clinical suspicion is high, and repeat testing at an appropriate interval is warranted. Repeat MAT performed 10 days after admission was positive (Figure 4).

The final diagnosis is Weil’s syndrome due to *Leptospira interrogans* serovar Icterohaemorrhagiae Copenhageni.

## Discussion

The genus *Orthohantavirus* includes viruses transmitted by rodents, causing a clinical spectrum ranging from asymptomatic infection to multiorgan failure. Two severe syndromic forms are described: hemorrhagic fever with renal syndrome (HFRS) and hantavirus cardiopulmonary syndrome (HCPS), the latter carrying 40-60% mortality and occasionally causing non-cardiogenic pulmonary edema with alveolar hemorrhage, although it remains rare in Europe. Diagnosis relies on serology or RT-PCR, and treatment is mainly supportive. Approximately 80 cases are reported annually in Belgium [7].

Leptospirosis is also a zoonosis, with rats as the main reservoir in Belgium; over the past five years, an average of 30 confirmed cases per year has been reported, according to Sciensano [4]. The pathogen is a Gram-negative spirochete, of which 22 species have been described, 10 pathogenic. Transmission occurs mainly through contact of broken skin or mucosa with water contaminated by infected rodent urine. The incubation period averages seven to 15 days (range 2-30), and the disease is classically biphasic, with an initial leptospiremic phase of approximately one week followed by an immune or leptospiruric phase [8].

Pulmonary involvement is among the most feared complications of leptospirosis. It may affect up to 70% of severe cases worldwide [5] and ranges from mild interstitial infiltrates to severe pulmonary hemorrhagic syndrome (SPHS) with diffuse alveolar hemorrhage, as observed in this patient. The pathogenesis of leptospiral pulmonary hemorrhage remains incompletely understood but is thought to involve direct toxin-mediated injury to pulmonary capillary endothelium, together with host immune-mediated mechanisms, resulting in capillary congestion, alveolar infiltration by red blood cells, and secondary ARDS [9]. Unlike hemorrhage related to classical pulmonary-renal syndromes, leptospiral alveolar hemorrhage is not primarily immune-complex or ANCA-mediated, which is consistent with the negative autoimmune work-up in this case. SPHS can develop with striking speed, sometimes within hours, as illustrated by the abrupt respiratory deterioration observed here, and it carries a reported mortality exceeding 50% despite intensive care support, with several series identifying pulmonary hemorrhage, rather than the classically emphasized hepatic or renal failure, as the leading cause of death in severe leptospirosis [6]. The infecting serovar may influence the clinical phenotype: icterohaemorrhagiae, identified in this patient, is among the serovars most consistently associated with Weil's disease and severe pulmonary hemorrhagic presentations, although serovar-specific virulence determinants are still incompletely characterized and formal serovar identification is available only in reference laboratories.

The pathophysiology of leptospirosis-associated diffuse alveolar hemorrhage differs fundamentally from that of classical immune-mediated pulmonary vasculitides. Histopathological studies of human hemorrhagic pulmonary leptospirosis have consistently shown capillary congestion and alveolar hemorrhage with only sparse inflammatory infiltrates [10], arguing against a primary vasculitic process driven by leukocytoclastic vessel-wall inflammation. Instead, the dominant mechanism appears to be direct, toxin-mediated injury to the pulmonary capillary endothelium: leptospiral outer-membrane components and putative hemolysins are thought to disrupt endothelial cell adherens junctions, increasing microvascular permeability and allowing red blood cells to extravasate into the alveolar spaces largely independent of a classical inflammatory cascade. This endothelial injury is compounded by host-derived factors, including local cytokine release and platelet-activating factor pathways, which may amplify capillary fragility and impair coagulation locally, even though thrombocytopenia and coagulopathy are not thought to be the primary drivers of hemorrhage. Alveolar epithelial cells themselves are relatively spared in early disease, with type I and II pneumocytes remaining largely intact, which may explain why some patients-including the present case-can achieve substantial pulmonary recovery once the acute hemorrhagic insult resolves. This “non-inflammatory” microvascular injury pattern, rather than a true pulmonary vasculitis, is an important conceptual distinction: it explains both the absence of the systemic vasculitic markers (ANCA, anti-GBM antibodies) sought and appropriately found negative in this patient, and the rationale for why immunosuppressive strategies effective in immune-complex-mediated alveolar hemorrhage (e.g., corticosteroids, cyclophosphamide) have shown inconsistent benefit in leptospirosis, where the injury is primarily toxin- and endothelium-driven rather than antibody-driven.

Diagnosis can be made by culture (blood during the first week, cerebrospinal fluid during weeks 1-2, and urine from the second week up to one month). Seroconversion usually occurs after the first week. MAT with ELISA confirmation is sensitive and specific, can be performed from the end of the first week, and remains reliable for up to one month, allowing serovar identification. PCR testing for Leptospira was not considered in this case, as this assay was not available at our institution. PCR has the advantage of detecting leptospiral DNA during the early leptospiremic phase, often before seroconversion occurs, and may therefore yield a positive result at a time when MAT is still negative, as illustrated by the diagnostic delay observed in this patient. Wider availability of Leptospira PCR in acute care settings could help shorten time to diagnosis in similarly severe presentations.

Several limitations of MAT are worth emphasizing, as they directly account for the diagnostic trajectory observed in this case. Because MAT detects agglutinating antibodies that typically only become measurable toward the end of the first week of illness, testing performed during the early leptospiremic phase-before seroconversion has occurred-frequently yields a false-negative result, as illustrated here. A single negative MAT obtained early in the disease course therefore cannot reliably exclude leptospirosis and should not be used in isolation to rule out the diagnosis when clinical suspicion remains high. For this reason, paired serology-an acute-phase sample followed by a convalescent-phase sample obtained one to two weeks later-is considered the reference approach, with a four-fold or greater rise in titer between the two samples providing the most robust serological confirmation, as ultimately observed in this patient between the initial and repeat MAT. Additional limitations include cross-reactivity between different Leptospira serovars, which can complicate serovar-specific interpretation, and the fact that antibody titers may wane over time, potentially yielding false-negative results if testing is delayed beyond the optimal window. These limitations underscore that MAT should be interpreted as a dynamic, time-dependent test rather than a single definitive result, and that repeat testing is warranted whenever the initial result is negative, but the clinical picture remains suggestive of leptospirosis.

Current guidelines converge on a relatively consistent approach to antimicrobial therapy in severe leptospirosis. The U.S. Centers for Disease Control and Prevention recommends intravenous penicillin G (1.5 million units every six hours) as first-line therapy for severe disease, with ceftriaxone (1 g intravenously every 24 hours) considered equally effective and often preferred for practical reasons, including single daily dosing and the absence of a need for penicillin allergy screening [11]. This equivalence is supported by randomized trial data showing comparable fever duration and mortality between ceftriaxone and penicillin G [12], as well as between cefotaxime, penicillin G, and doxycycline in a larger comparative trial [13]. A treatment duration of seven days is generally recommended for severe, hospitalized leptospirosis, in line with the regimen ultimately used in this patient once the diagnosis was confirmed. Importantly, all guidelines emphasize that antimicrobial therapy should be initiated empirically as soon as leptospirosis is clinically suspected, ideally within the first five days of illness, rather than delayed pending serological confirmation, given the nonspecific nature of early laboratory findings and the potential for rapid deterioration-an approach not possible in this case, where the diagnosis was only suspected after the acute respiratory crisis had already occurred.

Beyond antimicrobial therapy, current recommendations for severe leptospirosis center on early, aggressive organ support rather than any disease-specific intervention: lung-protective mechanical ventilation for ARDS, timely initiation of renal replacement therapy for oliguric or severe acute kidney injury, vasopressor support for distributive shock, and correction of coagulopathy and thrombocytopenia when clinically significant bleeding occurs. No specific guideline currently endorses corticosteroids, plasma exchange, or ECMO as standard of care for leptospirosis-associated pulmonary hemorrhage, given the absence of randomized controlled trial data; these remain individualized, case-by-case decisions reserved for refractory presentations, consistent with the approach adopted in this patient, who improved with supportive care and lung-protective ventilation alone.

Several recent publications underscore that severe pulmonary hemorrhagic leptospirosis remains a diagnostically challenging and highly lethal entity worldwide. Chen et al. [14] described a case in which metagenomic next-generation sequencing (mNGS) of bronchoalveolar lavage fluid allowed rapid identification of Leptospira in a patient with severe diffuse alveolar hemorrhage, highlighting the potential of this technique to shorten diagnostic delay when conventional serology is initially unrevealing. Yang et al. [15] similarly reported a case of pulmonary hemorrhagic leptospirosis complicated by invasive pulmonary aspergillosis, illustrating how atypical or co-infected presentations can further complicate diagnosis. Prieto Torres et al. [16] described a young patient with anicteric leptospirosis presenting with diffuse alveolar hemorrhage, reinforcing that severe pulmonary involvement can occur even without the classical jaundice of Weil's disease. Zhang et al. [17] reported successful use of veno-venous extracorporeal membrane oxygenation (VV-ECMO) in a patient with refractory hypoxemia due to leptospirosis-associated pulmonary hemorrhage, suggesting a potential rescue option in cases unresponsive to conventional ventilation.

Comparing the present case with these recently published reports highlights both recurring patterns and notable differences. As in the cases described by Chen et al. [14] and Yang et al. [15], our patient's initial serological work-up was unrevealing, and the diagnosis was only established after repeat testing or, in their cases, adjunctive molecular techniques-reinforcing that a single negative MAT should not exclude leptospirosis when clinical suspicion is high. Similarly to Prieto Torres et al. [16], our patient developed severe pulmonary hemorrhage in the absence of marked jaundice, underscoring that SPHS can occur across the icteric-anicteric spectrum of the disease and should not be discounted simply because classical Weil's disease stigmata are absent. Unlike Zhang et al. [17], whose patient required VV-ECMO for refractory hypoxemia, our patient recovered with lung-protective ventilation and multiorgan support alone, suggesting that ECMO, while a valuable rescue option, is not invariably required even in severe presentations, provided respiratory failure is recognized and treated promptly. A further distinction is the exposure setting: whereas several published cases originate from occupational or recreational exposure in endemic or rural environments, our patient's infection followed a brief accidental immersion in an urban canal in Western Europe, illustrating that clinically significant exposure can be incidental, easily overlooked, and geographically unexpected. Across these reports and the present case, the recurring clinical lessons are consistent: maintain a high index of suspicion for leptospirosis in unexplained multiorgan failure with pulmonary hemorrhage regardless of geographic setting or presence of jaundice, repeat serological testing when clinical suspicion persists despite an initial negative result, and consider molecular diagnostics or, in refractory cases, ECMO, as complementary tools rather than prerequisites for good outcomes.

Hantavirus and leptospirosis infections, which may present with nearly identical clinical pictures, remain a diagnostic challenge; in Belgium, a higher proportion of suspected leptospirosis cases show hantavirus IgM positivity than are serologically confirmed for leptospirosis.

This case emphasizes the importance of repeating etiological tests, as the definitive diagnosis was only obtained 10 days after admission. It also illustrates how a single detail from the clinical history can raise diagnostic suspicion, even for diseases usually considered endemic to tropical regions.

Collateral history is particularly valuable in critically ill patients with altered mental status, as encephalopathy-whether toxic-metabolic, infectious, or related to sedation and mechanical ventilation-often prevents the patient from providing a reliable exposure history when it is most needed. Relatives may be the only source of information regarding recreational, occupational, or environmental exposures that patients themselves fail to recognize as relevant or cannot recall, particularly when intoxication is involved. In this case, it was only through information volunteered by a family member, several days into the ICU stay, that the freshwater exposure came to light and prompted specific testing for leptospirosis. This highlights the value of systematic, proactive collateral history-taking in any critically ill patient with unexplained multiorgan failure and impaired consciousness.

Whether earlier recognition of the freshwater exposure could have meaningfully altered this patient's clinical course remains uncertain. Had the history of canal immersion been available at initial presentation, empiric antibiotic therapy would likely have included an agent with reliable activity against Leptospira, such as ceftriaxone, from the outset, and repeat MAT testing might have been pursued sooner, potentially shortening the diagnostic delay by several days. However, the abrupt onset of alveolar hemorrhage and ARDS occurred within hours of presentation, before any etiological result-positive or negative-could realistically have been available, and management of this complication would still have relied on the same supportive measures ultimately used: lung-protective mechanical ventilation, vasopressor support, and continuous renal replacement therapy, since no antimicrobial or targeted therapy is known to reverse established leptospiral alveolar hemorrhage. Earlier diagnosis may therefore have streamlined the diagnostic work-up and avoided some redundant or unnecessary testing, but it is unlikely to have changed the need for, or the intensity of, organ support during the acute respiratory crisis itself. Its main plausible benefit would have been earlier initiation of pathogen-directed antibiotic therapy, which is thought to reduce complications and bacterial shedding, and possibly closer anticipation of the risk of pulmonary hemorrhage, rather than a fundamentally different acute management strategy or a change in this patient's ultimately favorable outcome.

Despite presenting with severe pulmonary hemorrhagic syndrome, a complication associated with mortality frequently exceeding 50% in published series, this patient survived without recourse to rescue therapies such as ECMO, corticosteroids, or plasma exchange. Several factors may have contributed to this favorable outcome. First, respiratory deterioration was recognized immediately as it occurred in a monitored emergency department setting, allowing endotracheal intubation and transfer to intensive care without delay; series reporting the highest mortality often involve patients presenting later in the course of respiratory failure or in settings with delayed access to mechanical ventilation. Second, a lung-protective ventilation strategy with high PEEP and low tidal volumes was applied promptly, limiting secondary ventilator-induced lung injury on top of the primary hemorrhagic insult. Third, aggressive multiorgan support-vasopressors, continuous renal replacement therapy, and broad-spectrum antimicrobial therapy-was instituted early, even before the causative organism was identified, addressing the multiorgan failure that itself contributes substantially to mortality in severe leptospirosis. Fourth, the patient was relatively young and, aside from his cardiovascular history, had no chronic pulmonary or immunosuppressive condition that might have compounded the severity of ARDS. Finally, once the diagnosis was confirmed, targeted antimicrobial therapy could be instituted, potentially limiting ongoing bacterial toxin-mediated endothelial injury. While the relative contribution of each factor cannot be individually quantified in a single case, this presentation illustrates that survival after severe leptospirosis-associated pulmonary hemorrhage is achievable with prompt recognition of respiratory failure and aggressive, protocolized intensive care support, even when specific antimicrobial therapy is delayed by diagnostic uncertainty.

## Conclusions

Leptospirosis can rapidly progress from nonspecific symptoms to life-threatening multiorgan failure and alveolar hemorrhage. In non-endemic settings, diagnosis relies heavily on clinical suspicion, as initial tests may be negative. Early empiric treatment and careful assessment of exposure history are critical to improving patient outcomes. This case highlights three practical clinical messages for physicians managing patients with unexplained multiorgan failure. First, a high index of suspicion for leptospirosis should be maintained even in non-endemic, temperate regions such as Western Europe, as incidental freshwater exposure can occur in everyday urban settings and should not be dismissed on the basis of geography alone. Second, an initial negative MAT does not exclude the diagnosis: serological testing should be repeated when clinical suspicion persists, particularly if the first sample was obtained early in the disease course, before seroconversion has occurred. Third, when patients are unable to provide a reliable history due to altered mental status, confusion, or sedation, clinicians should proactively and systematically seek a detailed exposure history from family members or other collateral sources, as this information may be the only clue capable of directing appropriate etiological testing.
